# Supplementary figures and images for: Haem transporter HRG-1 is essential in the barber’s pole worm and an intervention target candidate
Source: PLoS Pathog. 2023 Jan 30;19(1):e1011129. doi: 10.1371/journal.ppat.1011129 (PMC9910794; doi:10.1371/journal.ppat.1011129)

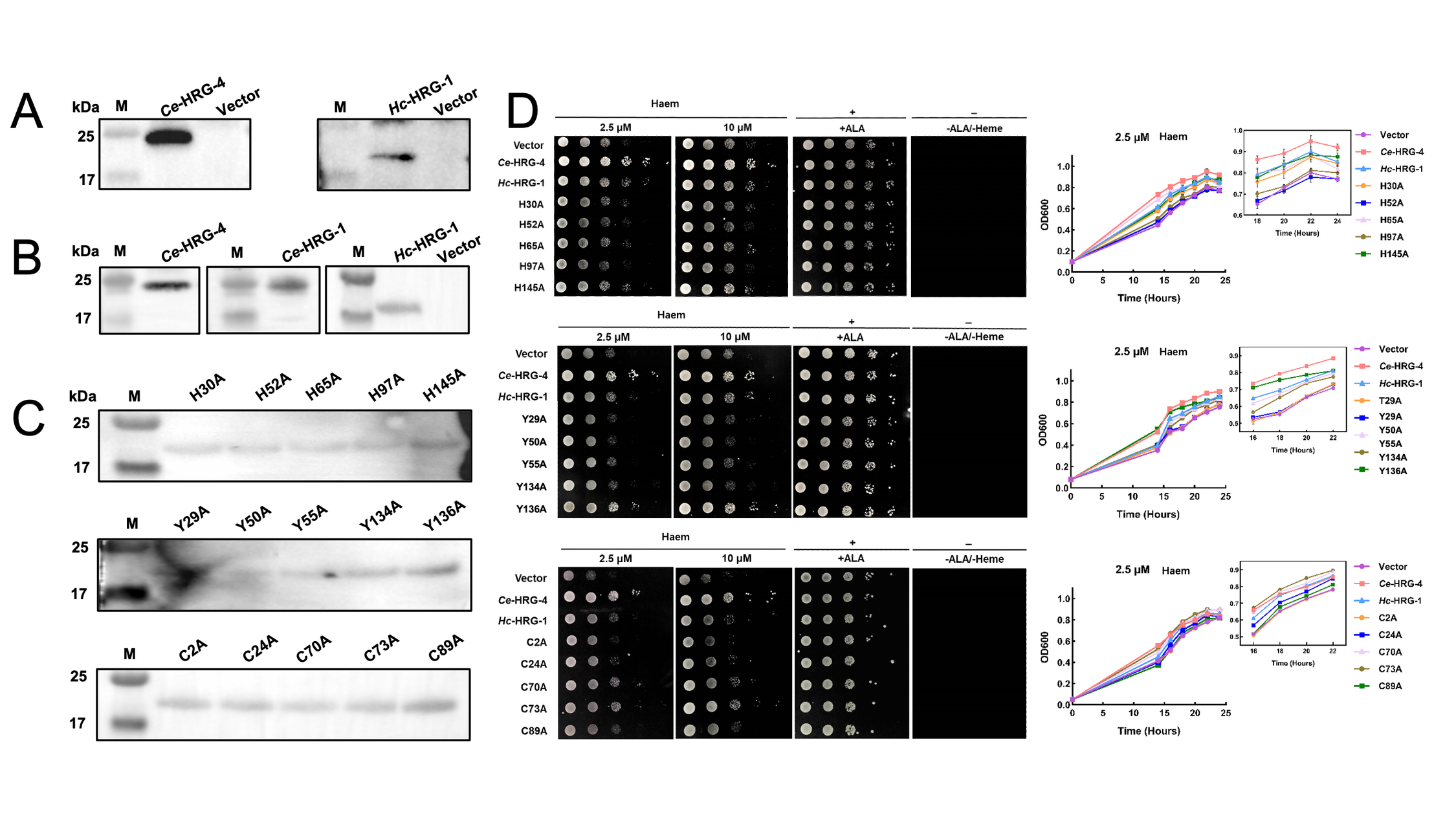

Supplement: S1 Fig — (A) Heterologous protein expression of FLAG-tagged Ce-HRG-4 or Hc-HRG-1 in Δhem1 yeast strain verified by Western blot analysis. (B) Heterologous protein expression of FLAG-tagged Ce-HRG-1/4 or Hc-HRG-1 in BY4741 yeast strain verified by Western blot analysis. (C) Heterologous protein expression of FLAG-tagged Hc-HRG-1 (H30A, H52A, H65A, H97A, H145A, Y29A, Y50A, Y55A, Y134A, Y136A, C2A, C24A, C70A, C73A and C89A) mutants in Δhem1 yeast. (D) Spotting assay and growth curve of yeast transformed with empty vector, Ce-HRG-4, Hc-HRG-1 or Hc-HRG-1 mutants with or without supplementation with haem (2.5 μM or 10 μM) or 5-aminolevulinic acid (ALA–an intermediate in the haem biosynthesis). M: protein molecular weight marker. (TIF) [file ppat.1011129.s003.tif]

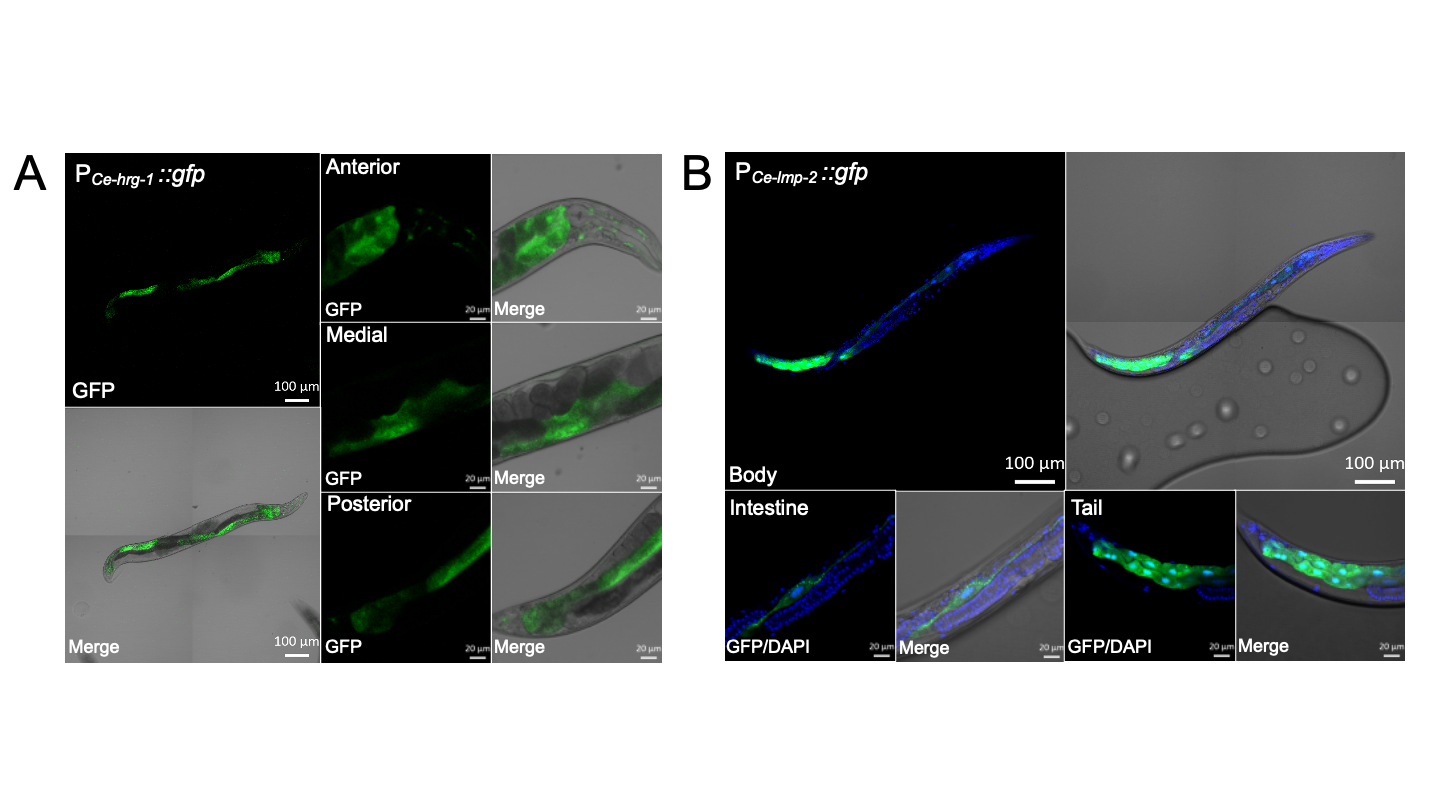

Supplement: S2 Fig — (A) Prediction of the HERE for Hc-hrg-1 based on information on the haem responsive genes (e.g., Ce-hrg-1 and Ce-hrg-7) from the free-living model organism Caenorhabditis elegans. (B) An image showing the ensheathed infective larvae of H. contortus that is transcriptionally non-responsive to haem supplementation. Sheath is indicted by red arrows. (TIF) [file ppat.1011129.s004.tif]

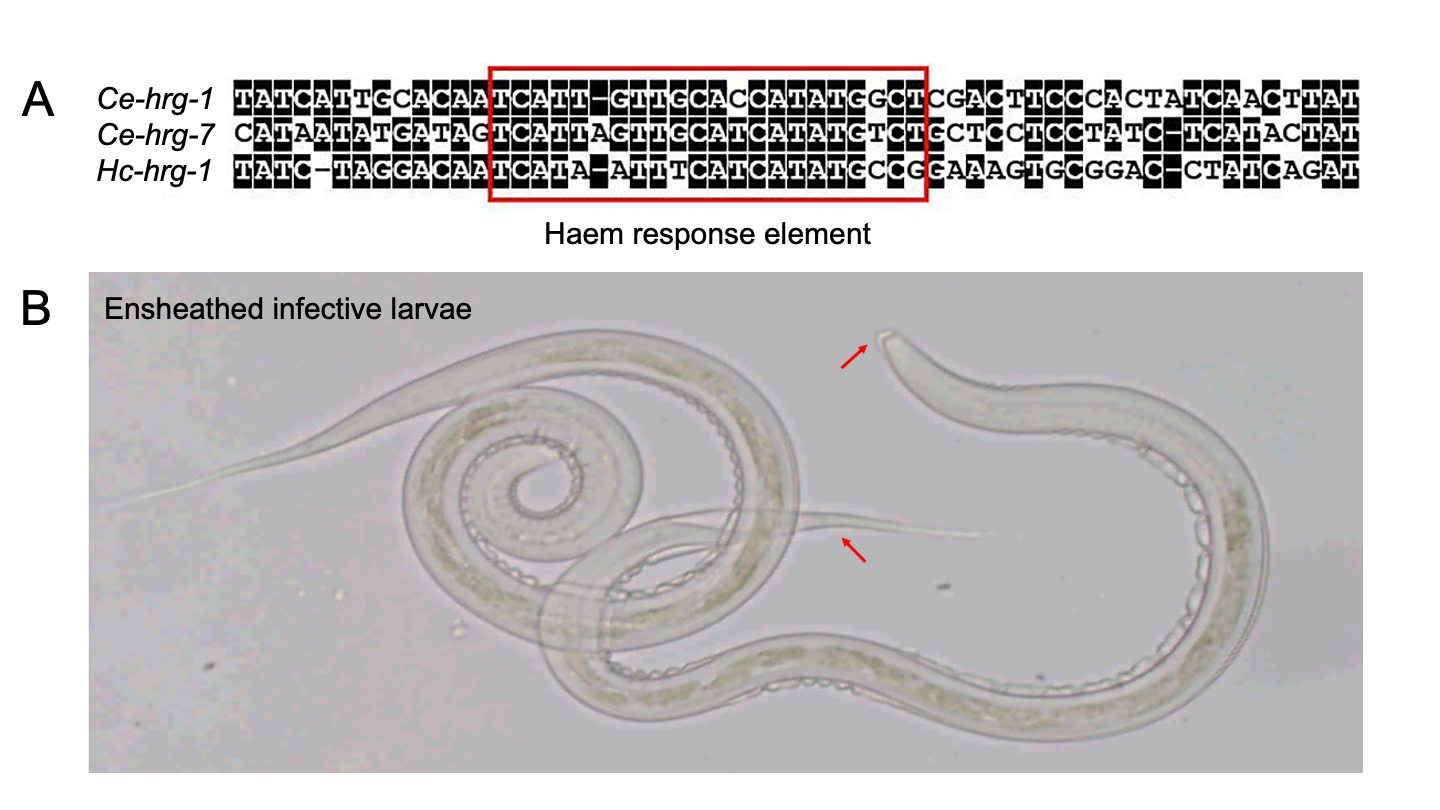

Supplement: S3 Fig — (A) Promoter of hrg-1 (PCe-hrg-1) drives the expression of green fluorescent protein coding gene (gfp) in the anterior, medial and posterior intestine of C. elegans. (B) Promoter of lysosomal associated membrane protein 2 coding gene (PCe-lmp-2) drives gene expression in the posterior intestine of C. elegans. GFP: green fluorescent protein. DAPI: 4’,6-diamidino-2-phenylindole. (TIF) [file ppat.1011129.s005.tif]
