# Supplementary material for: Haem transporter HRG-1 is essential in the barber’s pole worm and an intervention target candidate
Source: PLoS Pathog. 2023 Jan 30;19(1):e1011129. doi: 10.1371/journal.ppat.1011129 (PMC9910794; doi:10.1371/journal.ppat.1011129)
Supplement: Attachment — Submitted filename: PPATHOGENS-D-22-01671_Rejoinder.docx [file ppat.1011129.s007.docx]

**9 January 2023**

**Professor Edward Mitre**

*Academic Editor of PLOS Pathogens*

**Professor P'ng Loke**

*Section Editor of PLOS Pathogens*

**Professor** **Kasturi Haldar**

*Editor-in-Chief of PLOS Pathogens*

**Professor** **Michael Malim**

*Editor-in-Chief of PLOS Pathogens*

**Dear Professors Mitre, Loke, Ksturi Haldar and Malim,**

**RE: Manuscript reference number: PPATHOGENS-D-22-01671; *Title: Haem transporter HRG-1 is essential in the barber’s pole worm and an intervention target candidate.***

**Sincere thanks for handling our manuscript. We are grateful to the referees for their reports. In the following, please find a detailed rejoinder that addresses the reviewers’ issues in a point-by-point manner (authors’ responses in bold-type).**

**EDITOR’S COMMENTS**

**E1.** The manuscript was reviewed by three outside experts. All reviewers felt that the work is important and evaluates an important issue (haem uptake) in Haemonchus contortus. In addition to providing insights on the biology of this nematode, the finding of the essential nature of HRG-1 indicates a possible novel drug target for H. contortus and perhaps other nematodes as well. That stated, the reviewers raise several issues that affect how well the study ultimately proves the conclusions. If you are able to address these issues in a timely manner, the revised manuscript will be re-considered for publication. If this is not possible, would consider submitting to another journal.

**Response: Thank you for your positive and helpful comments, and for the opportunity for us to revise our manuscript. We appreciate the detailed and critical reports from the reviewers; all of the issues have been addressed.**

**E2.** While all the comments from the reviewers should be addressed in a possible response, it would be critical to address the following concerns:

- Describe the in vitro binding data in the text.

**Response: Thanks. Details on haem binding assay have been provided in the text (lines 109-112).**

**E3.** In figure 2B, please show (or mention in results) objective data of transformant growth when grown with ALA, and please show growth of normal (Wild-type) yeast alongside transformants with and without ALA supplementation.

**Response: Thanks. The growth of normal (wild-type BY4741) yeast is shown in Figure 2A (right panel) with and without ALA supplementation, to verify the haem-deficient strain. We did not include wild-type yeast alongside *Ce*-HRG-1/4 and *Hc*-HRG-1 transformants in the subsequent experiments (Figure 2B and 2C), because yeast transformed with an empty vector is the appropriate control. Regarding transformant growth with ALA, this is shown in Figure 2B (positive panel), revealing a markedly better growth than vector, *Ce*-HRG-4 and *Hc*-HRG-1 transformants.**

**With due respect, we do not agree with the comment from Reviewer #3 that “***The Hc-HRG-1 transformant doesn't look very different to my eyes from the empty vector.***”, as there are distinct differences (particularly when supplemented with highly-diluted haem) in the growth of yeast transformed with empty vector and HRGs (see high-resolution image). Nonetheless, we quantified the growth of transformants, based on a growth curve, which is shown in S1 Figure. We have also slightly modified our description in the text to be more objective (lines 115-117).**

**E4.** Show data that transformants with targeted mutations exhibit decreased growth. Currently the line stating this refers to figure 2D, which only shows a structural diagram.

**Response: We are grateful for your observation. We have addressed this issue by referring our statement to S1 Table.**

**E5.** Improve labeling in figure 7.

**Response: Nematode a have simple but characteristic structure, particularly for the intestinal and reproductive tracts in a cross section of worm. More labels have been provided to indicate the position of intestinal and reproductive tracts in the indirect immunofluorescence assay (IFA) images and schematic diagrams for *H. contortus*.**

**E6.** Add to methods the sequences used for cloning, qPCR, and siRNA experiments

**Response: Sequences for primer sets used in our work are listed in S2 Table, as stated in the text. We have modified the Method section and cited relevant references to make this more evident.**

**E7.** Add legends for the supplemental figures

**Response: Legends for the supplemental figures were provided in Supporting information section of the original manuscript.**

**E8.** Expand the discussion to place the work in the context of prior studies.

**Response: Thanks. The *Discussion* section has been expanded to place our novel findings in the context of previous studies in *C. elegans* and *Brugia malayi*, particularly in relation to issues and limitation raised by the reviewers.**

**E9.** Given that the worms used to infect sheep were already dying at time of infection, the relevance of in vivo challenge of sheep is a bit unclear. At a minimum, would add language clearly stating the limitation of this experiment.

**Response: Thanks. Limitation of the animal experiment and related issues raised by the reviewers have been discussed (lines 277-289).**

**REVIEWER #1**

**Part I - Summary**

**1.1.** Reviewer #1: The manuscript by Yang and coworkers describes studies to characterize the function of a heme transporter in Haemonchus contortus. The work is thorough and important. The manuscript is well written.

**Response: Thank you for your positive and helpful comments.**

**Part II – Major Issues: Key Experiments Required for Acceptance**

N/A

**Part III – Minor Issues: Editorial and Data Presentation Modifications**

**1.2.** More explanation of methods used in each part of the results would be useful. Not detailed methods, but a general overview of what was done. For example, line 109, it is stated that Hc-hrg-1 binds heme in vitro; explain how this was determined?

**Response: We are grateful for these queries. More details on the methods used have been provided in the revised manuscript (lines 109-112, 183, and 198, etc.).**

**1.3.** Line 113: the Hc-HRG-1 transformed cells maybe grew a little better than dhem1 cells alone, but not nearly that of Ce-HRG-4 transformed cells. Why was Ce-HRG-4 and not HRG-1 used? The GaPPIX treatment was more convincing.

**Response: In *C. elegans*, the canonical movement of haem within a cell is usually mediated by HRG-4 (in the apical plasma membrane) then HRG-1 (in the subcellular compartment). Therefore, *Ce-*HRG-4 was used (rather than *Ce-*HRG-1) in the haem acquisition assay in *Δhem1* cells. We have modified the wording to make this clearer in the text (lines 115-117). Thanks for your positive comment on the GaPPIX treatment assay.**

**1.4.** Line 121, mutagenesis: Were the mutant proteins expressed and nonfunctional or simply not expressed or unstable?

**Response: Mutant proteins were successfully expressed in yeast and verified by Western blot analysis (see S1 Figure in the revised manuscript). Yeast transformed with *Hc*-HRG-1 mutants (C2A, Y29A, Y50A, H52A or H97A) appeared to be not functional in haem acquisition.**

**1.5.** The yeast 2-hybrid screen identified only the V-ATPase and no other binding partners?

**Response: No. We identified several proteins that might interact with *Hc*-HRG-1, but only the V-ATPase 2 was positive in the reverse Y2H and subsequent assays.**

**1.6.** Comment on the L3 having highest Hc-hrg-1 mRNA, but exogenous heme was not accessible due to ensheathment. What is the function of Hc-hrg-1 in this stage?

**Response: Thanks for your comments. We have shown (in other studies) that there is a remarkable storage of mRNAs in ensheathed L3s of *H. contortus*. A hypothesis of pre-adaptation to environmental changes after entering the host animals has been proposed for this worm in this and previous work (Ma et al., Int J Parasitol, 2018; Ma et al., Adv Parasitol, 2020). Current evidence also indicates that high transcription of *Hc-hrg-1* in the L3s stage is a pre-adaptation to the infection.**

**1.7.** Line 166: The way I read this Hc-hrg-1 RNA was used to silence Ce-hrg-1 in Ce. If there is similarity between Hc and Ce this is expected. What about Ce-hrg-1 paralogues in Ce? Would these also be silenced by Hc-hrg-1 treatment? Is this the same result seen in Ce silencing with Ce sequences?

**Response: Thanks for your comments. There are three paralogues (*hrg-4, -5* and -*6*) to *hrg-1* in the free-living nematode *C. elegans*. However, only one haem transporter-coding gene was identified in *H. contortus* and also in a range of parasitic nematodes, which consistently showed highest identity to the *Ce-hrg-1*. Therefore, we used *Hc-hrg-1* RNA to silence *Ce-hrg-1* in *C. elegans* (heterologous RNAi). However, we did not determine whether the transcription of *hrg-4, -5* or *-6* was affected by the *Hc-hrg-1* RNAi, because of their distinctiveness in sequence and function in *C. elegans*. The same phenotype resulting from *hrg-1* RNAi in *C. elegans* has been reported previously.**

**1.8.** Line 174: Silencing both hrg-1 and vha-1 lead to an increase in hrg-1 mRNA?

**Response: Yes. We found that RNAi of *vha-2* significantly increased the transcriptional level of *hrg-1* in *H. contortus*, likely due to a feedback circuit (which has not yet been defined). To verify this finding, we conducted RNAi of *hrg-1* and *vha-2* simultaneously, which markedly compromised the RNAi-mediated gene knockdown of *hrg-1* in the treated worms. We proposed a possible feedback circuit for *hrg-1* and *vha-2* (see Figure 8K), which will warrant further investigation.**

**1.9.** Line 187: It is indicated that heme in excess is toxic. How does Hc deal with excess heme?

**Response: We performed haem-responsive transcription analysis of *hrg-1* in *H. contortus*. By supplementing haem to the culture medium, we detected a downregulation of *hrg-1* in treated worms, inferred to lead to a reduced uptake of haem. Additionally, haem detoxification mechanisms have also been reported in parasitic nematodes (Bouchery et al., PLoS Pathog, 2018).**

**1.9.** Line 203: Please explain how adding exogenous heme rescues worms that have had the protein that acquires exogenous heme silenced.

**Response: Due to well recognised technical challenges (please see Zawadzki et al., Trends Parasitol, 2006; Geldhof et al., Int J Parasitol, 2006; Samarasinghe et al., Int J Parasitol, 2011; Britton et al., Adv Paraitol, 2016), RNAi-mediated gene knockdown does usually not achieve complete gene knockdown in *H. contortus* and other parasitic nematodes. Please also see response to point 1.8. Therefore, RNAi of *hrg-1* resulted in a reduced transcriptional level of this gene rather than deficiency of the protein in the treated *H. contortus*. The reduced abundance of HRG-1 in RNAi-treated worms could be reasonably/partially rescued by supplementing exogenous haem.**

**1.10.** Ce has 4 paralogues of hrg-1. These should be discussed in more detail.

**Response: Information on paralogues of *hrg-1* in *C. elegans* is provided in both the *Introduction* and *Results* sections of our original manuscript. We have now provided more information on the *hrg-1* paralogues in *C. elegans* and the uniqueness of *hrg-1* in *H. contortus* and other parasitic nematodes in the Discussion section. Please also see our response to point 1.7.**

**1.11.** Fig. 8. E and F are after 3 d treatment and G and H after 7 ay treatment? Sick + dead after 7 d treatment is about 60%. These were used to infect sheep and patent infection was not observed. Is this surprising since most of the worms were already dead or dying? This questions the usefulness of the in vivo studies.

**Response: Yes. Thanks for your comments. There are challenges in precisely determining phenotypes resulting from RNAi treatment in *H. contortus* and other parasitic nematodes. Larval motility and development assays have been conducted in previous studies, and can be used to some extent to quantify these phenotypes. However, these *in vitro* assays cannot directly verify the functional essentiality of a gene. Therefore, an infection experiment using RNAi-treated larvae should be preferably conducted. Although ~ 40% of the treated larvae appeared normal, they did not establish infection or survive in their host animals (in a controlled experiment). Limitations of such animal experimentation have been discussed in the revised manuscript (lines 283-284).**

**1.12.** Discussion of the results of the study was only 2 paragraphs. This should be expanded. Place the results in context of previous studies. For example, how do the results of this study compare to the study of heme uptake/transport in Ce and Brugia?

**Response: As required for publication in a high impact journal, such as PLoS Pathogens, we wanted to be as concise in our discussion as possible. Previous knowledge about haem uptake/transport in *C. elegans* and *Brugia* species has already been compared/linked/cited in the *Results* section. The manuscript has been revised (see track changes); we have elected only to discuss pertinent issues/limitations, and have now included issues raised by the reviewers and editors which were not covered in the original version.**

**1.13.** Are there legends for the supplemental figures/table? Was is the meaning of Target %id and Query %id in SI table (2)?

**Response: Yes. Legends for supplemental figures and tables are provided in the Supporting information section of the original manuscript. In this work, a reciprocal best Blast searching approach was performed to identify *hrg-1* orthologues in a range of parasitic nematodes (clades V, IV, III and I) of animals. The “Target %id” was the identity of an identified sequence against that of *Ce-hrg-1*, whereas the “Query %id” was the identity of the identified sequence against that of a best hit in *C. elegans* database (namely *Ce-hrg-1*). We have provided a footnote for these subtitles in S1 Table.**

**REVIEWER #2**

**Part I - Summary**

**2.1** The article by Yang et al describes the role of the heme transporter HRG-1 is Haemonchus contortus using molecular approaches in yest, C. elegans and H.contortus itself. Given nematodes can not de novo synthetise heme but need to acquire it from their environment, HRG-1 is a potential target of interest for sustainable control of Hc. The authors showns that nematode parasite have only one HRG-1, that it is associated with endosome/lysosome and that RNAi decrease parasite survival. Alltogether, there is substantial evidence that moving forward HRG-1 as a drug or vaccine target could be of interest.

**Response: We are grateful to your positive and helpful comments.**

**Part II – Major Issues: Key Experiments Required for Acceptance**

**2.2.** Line 188 : This point is in apparent contradiction with figure 6A, where the expression is found to be higher in males in the adult stage. Interestingly the data are not provided for adult females. Rather than having this comment based on one representative image, it would be interesting to have an actual quantification on HRG1 expression in gonads of male and female to ensure that this point is correct. Of note, in the male section, the intestine expression of HRG1 also seems much weaker than in the female, the difference might thus more due to an suboptimal choice of representative image.

**Response: We respectfully disagree with the comment that our statement is “in apparent contradiction”. Figure 6A shows the relative transcriptional levels of *hrg-1* in female and male adults of *H. contortus*, whereas Figure 7B indicates the distribution of HRG-1 in female and male adults of this nematode. We did not find any statement in our manuscript relating to your statement “***the expression is found to be higher in males in the adult stage***”, such that we have not addressed this: “***ensure that this point is correct***”. There must have been a misunderstanding. We did not compare the abundance of HRG-1 between the sexes of *H. contortus*, and we do not think that it is informative to do this based on relative mRNA levels of a gene between sexes and tissues (typically fluorescence intensity), as tight post-transcriptional regulation has already been reported in this parasitic nematode (Ma et al., Int J Parasitol, 2018).**

**Additionally, we did not understand the statement “**Interestingly the data are not provided for adult females.**”, since results of gene transcription and protein distribution in the female and male adult worms have been described/shown in the text and figures. The immunofluorescent assay figures were provided to indicate the tissue distribution of HRG-1 in *H. contortus*, not for a comparison between female and male adults.**

**2.3.** For figure 7: It would have made comparison between parasites much easier to have similar staining presented (section versus whole worm). I am in particular not sure whether the authors can claim a "basal laminae" staining based on those images. Why would it be "apical membrane" for Ce and "basal laminae" for Hc for intestine? The intestinal staining in Hc certainly looks in the intstinal epithelium itself, as it is quite diffuse around the nuclei of the intestinal epithelium. Due to high autofluorescence of many nematode tissues, the authors should present images of non GFP parasite to confirm specificity of HRG-1 localisation

**Response: As described (lines 183-187), HRG-1 exhibited different tissue/cellular localisation in *C. elegans* (reported in intestinal apical membrane and intracellular) and *H. contortus* (found predominantly in basal laminae and cell membrane). This discrepancy can be explained by the multiple HRG-1 paralogues in *C. elegans.* We agree with your comments about the autofluorescence in nematode species, and that was why we did not focus on the diffuse fluorescence in the intestinal epithelium. The fluorescence signal was much stronger in the basal laminae in the parasitic nematode studied. In addition, the GFP-based fluorescence assay was performed only in *C. elegans*, while indirect immunofluorescence chemistry was performed in *H. contortus* due to technical limitations relating to most parasitic nematodes. We have slightly modified our description to prevent an overinterpretation of the findings.**

**Part III – Minor Issues: Editorial and Data Presentation Modifications**

**2.4.** The english is not always clear. In particular lengthy sentences, make understanding very difficult. Please see attached document to see details of which sentence need changes.

**Response: The manuscript has been scrutinised and improved.**

**2.5.** Figure 1A : What species have actually been investigated ? Were there any that did not have HRG-1 homologues ? It would of interest if Figure S1 could answer those questions

**Response: We investigated 41 species of parasitic nematodes. Most species have one *hrg-1* gene, whereas two copies of the *hrg-1* gene were predicted for *Ancylostoma caninum* and *Dirofilaria immitis*. Information on the species and gene orthologues in these parasitic nematodes is provided in the S1 Table (original manuscript).**

**2.6.** line 99-101 : the authors describe that HRG-1 homology to human orthologues is limited, but pointed out that "particular amino acid (aa) residues in sequences linked to haem transport were invariable among the nematode and mammalian species" --> could the authors please speculates on whether this would be a problem for targeting HRG-1 for example for vaccine design.

**Response: Considering the function of HRG-1 (haem transport), it is not surprising to us that amino acid residues associated with haem binding are conserved among nematodes and mammalian species. Nonetheless, the limited sequence homology of HRG-1 between nematodes and host animals (overall) means that a variable fragment of nematode HRG-1 might be targeted by antibodies or interfering RNAs for intervention.**

**2.7.** Line 153: I do not understand the cause/effect link the authors are referring to. It would be help to rephrase/explain more clearly what the authors mean.

**Response: Vacuolar H(+)-ATPases (VHAs) are well-known component of endo-lysosomes (or lysosome-related organelles) in metazoa. The interaction of HRG-1 with VHA-2 in *H. contortus* indicates an involvement of the endo-lysosomal system in haem trafficking *via* HRG-1 in this parasitic nematode. We have slightly modified this sentence to make this clearer (lines 157-158).**

**2.8.** Line 166: please define what the "Haem aquisition model" is.

**Response: Thanks. Addressed.**

**2.9.** Figure 6a : The authors speculate that the high transcription of HRG-1 after heme exposure in L3 is linked to unresponsiveness to heme as L3 is a closed stage. However, I do not understand why it is so high in L3 and not in other free living stages. To me it looks like it is massively produced at the infective stage as it is highly required early on for infection ? It is really easy in vitro to activate iL3 with CO2 and 37C, and it would be interesting to see if the parasitic L3 stage is more similar to the infectious or to L4 to understand the role of HRG-1, as the authors later claim in the paper that "knock down" of HRG-1 prevent parasite establishment.

**Response: Indeed. Based on our previous work (Ma et al., Int J Parasitol, 2018; Ma et al., PLoS Pathog, 2019), there is a remarkable storage of mRNAs in ensheathed L3s of *H. contortus*. A hypothesis of “pre-adaptation to environmental changes after entering the host animals has been proposed for this and related parasitic nematodes”. In the previous work, we showed that mRNAs of *hrg-1* and many other genes were present at high levels, and low levels in activated (exsheathed) L3s *in vitro*.**

**2.10.** Line 179: unclear sentence. Too long. The figure 7A and B are hard to compare giving the difference in cross section versus entire worm. It is in particular unclear to me that the HRG-1 expression is present in the basal lamina. I see a clear stain of the intestinal epidermis. I think the resolution presented here do not allow to distinguish staining of the epithelium versus the basal lamina that border the thin epithelium. The authors should mitigate their claim or provide evidence for staining of the lamina only. Notably, it would be good here to provide section of non reporter H. contortus to ensure the specificity of the green signal. Many structure of nematode are naturally autofluorescent.

**Response: Thanks. Please also see our responses to comments 2.2 and 2.3. Thanks.**

**2.11.** Lines 182-187: This should be added to discussion rather than the result section as it is more speculative.

**Response: We would argue that these sentences are essential to describe the tissue expression patterns of HRG-1 in *C. elegans* and *H. contortus*, and important links to the studies on HRG-1-mediated haem acquisition and homeostasis in the latter species. We have also discussed this aspect (lines 280-283).**

**2.12.** Figure 8: I respectfully disagree with the authors interpretation of the data presented. Please justify/discuss this point and perform appropriate statistical test (see comments in the pdf line 194 and 203)

*Line 194: I do not agree with the description/interpretation of these results. Despite the high mortality rate of the HRG1 RNAi control (no GaPPIX), the addition of GaPPIX still increase substantially the death as it does in control Hc individuals.*

**Response: Thanks for your comments. It would be easier to understand these results by considering the RNAi efficiency (usually low) in parasitic nematodes. *Hc-hrg-1* was not completely silenced, and the retained mRNA would translate as protein and function in haem transport (essentially GaPPIX transport here), leading to the death of worms at an earlier stage (days 2 and 3) of incubation with 5 or 10 µM GaPPIX. RNAi-mediated gene knockdown takes time, and it was clearly shown/described that efficient knockdown of *Hc-hrg-1* significantly reduced the toxic effect of 5 µM and 20 µM of GaPPIX (a toxic haem analogue) on larvae (P < 0.05) after four days of treatment, compared with control. Please see our response to comment 1.9 as well.**

*Line 203: For this claim the statistical comparison presented are not the right one, you would need o versus 5 or 10 uM for the control and RNAi.*

**Response: We are grateful for this comment. We have critically revised our description/statement to avoid misunderstanding or overinterpretation of the experimental design and results.**

**2.13.** Line 218: Nothing in the experiment presented can point out that the lower adult worm/egg outpout burden at endpoint is linked to a decrease establishment of parasite. It could either be lower establishment or lower survival/reproduction. Earlier time point is required for making this claim. Please rephrase accordingly or add the new experiment.

**Response: Thanks for your critical comments and suggestions. We have altered the title and related elements in the manuscript.**

**REVIEWER #3’**

**Part I - Summary**

**3.1.** This is important work that focuses on a still understudied parasite despite its veterinary importance. I liked aspects of the study but I found major issues as well.

STRENGTHS: Much of the study appears novel, and represents an ambitious attempt to study haem uptake in Hc in some detail. In particular the animal experiments are compelling.
WEAKNESSES: I have concerns around execution and interpretation of some of the data, as described in more detail below.

**Response: Thank you for your positive and helpful comments. We have critically scrutinised our manuscript and made revisions based on your suggestions.**

**Part II – Major Issues: Key Experiments Required for Acceptance**

**3.2.** LINE 109: Refers to in vitro experiments as already having been completed, without any previous description of these or presentation of data. Associated Figure 1D shows in vitro binding data but these data are not described in the results text.

**Response: We have provided results for the haem binding assay (lines 109-112).**

**3.3.** LINE 113: "Grew normally" - is a dubious claim. The Hc-HRG-1 transformant doesn't look very different to my eyes from the empty vector. I would ask for these experiments to include a wild type +ve control to assess whether growth is "normal". Evaluation of these results as significant is currently subjective; yeast growth images need to be quantified by densitometry or similar, with statistical analysis of data.

**Response: We would argue that there are obvious differences (particularly when supplemented with highly diluted haem) in the growth of yeast transformed with empty vector and HRG in the high-resolution image. Growth of normal (wild-type BY4741) yeast is shown in Figure 2A (right panel) with and without ALA supplementation to verify the haem-deficient strain. We did not include wild-type yeast alongside *Ce*-HRG-1/4 and *Hc*-HRG-1 transformants in subsequent experiments (Figure 2B and 2C), because yeast transformed with empty vector is considered the appropriate control. We quantified the growth of transformants based on their growth curve, shown in S1 Figure.**

**We have slightly modified our description in the text to make our statement more objective and evident, and to avoid misunderstanding.**

**3.4.** LINE 123: Claims that transformants carrying targeted mutations exhibit decreased growth, referring to Figure 2D. Figure 2D is merely a structural diagram, so the data supporting this claim is not shown.

**Response: Thanks for pointing this out. We have provided and referred to a supplementary figure (S1 Fig) in the revised manuscript to support our statement.**

**3.5.** LINE 130: Co-localisation of HRG/ZnMP refers to Fig 3B subpanel. Red fluorescence in these panels is not clear, making it difficult to interpret the co-localisation claim.

**Response: We acknowledge that red fluorescence in the longitudinal section of *C. elegans* might not be optimal. Therefore, zoom-in subpanels were provided for a better appraisal (GFP and ZnMP). We would argue that the red fluorescence in these subpanels is clear, and the Figure 3 was converted by the Preflight Analysis and Conversion Engine (PACE) digital diagnostic tool, which meets PLOS requirements.**

**3.6.** LINE 167: States that "In this C. elegans model, Hc-hrg-1 RNA interference (RNAi)-mediated (heterologus) gene knockdown resulted in a significant (P < 0.01) decrease in the Ce-hrg-1 mRNA level, being comparable to homologous knockdown of Ce-hrg-1 (P < 0.01) (Fig 6C)", which is a confusing statement. Does this imply that the Ce were expressing Hc-HRG? Or that Ce were treated with Hc-HRG dsRNA? This section needs to be written more clearly. AS a non-specialist it is not clear to me why HRG knockdown INCREASES ZNP fluorescence? Shouldn't this impede ZNP uptake leading to REDUCED fluorescence? Again, this needs to be more clearly explained.

**Response: Thanks for your comments. Heterologous RNAi is a strategy widely used in the free-living model organism *C. elegans* to infer the function of a gene in parasitic nematodes, for which limited reverse genetic tools are available. ZnMP was not imported into the intestine and (then) other tissues of *C. elegans* when the transporter was supressed, leading to an increased signal in the intestine tract. We have modified the description in the text to make these aspects clearer.**

**3.7.** LINE 181, and Figure 7B: How do we know Hc localisation is gonadal? Clearer labelling and/or additional evidence is required to demonstrate that the observed fluorescence is associated with reproductive tissue. Also the diagram in Figure 7D could be useful in this regard but currently doesn't illustrate reproductive expression at all.

**Response: Thanks for your comments. Nematodes have simple but characteristic structures, particularly for the intestinal and reproductive tracts. We have provided more labels in the Figure 7B and D to make them clearer.**

**3.8.** MOLECULAR METHODS: No primer sequences have been defined, making replication of cloning, qPCR and RNAi experiments impossible.

**Response: Detailed information on primer sets used for cloning, qPCR, RNAi and other experiments is provided in the S2 Table of the original manuscript. We have also cross-referenced this in the text.**

**3.9.** Fig 5 legend: Meaning of "HA" in Hc-HRG-1-HA is not defined.

**Response: Thanks. We have defined abbreviations upon first usage.**

**3.10.** LINE 199: Refers to RNAi phenotypes including "dead" and "sick" worms. These are subjective terms, and should be quantified and explained more clearly.

**Response: Phenotypes such as “dead (lethal)” and “sick (some combination of abnormal features relating to size, movement, body integrity, pigmentation, viability, and/or fertility, etc.)” are commonly used in the RNAi research of nematodes. Sick is defined as “low vitality, developmental retarded or deformed” in the text of our original manuscript. Regarding the comments on quantification, the motility assay was established to quantify these phenotypes, but these phenotypes need to be manually verified and summarised as lethal or sick for technical reasons. The text has been slightly modified to address this issue, but the original description was considered clear and correct. Thanks.**

**Part III – Minor Issues: Editorial and Data Presentation Modifications**

N/A

**CONCLUSION**

**In conclusion, we thank you and the reviewers for their time, and their detailed and constructive reports. We have addressed all issues raised, in a point-by-point manner. We believe that we have thoroughly addressed the comments, criticisms and/or suggestions. We elected to make some minor alterations to text, figures and legends to further enhance the quality of the revised manuscript.**

**We have submitted two versions (“tracked” and “clean”) of the revised manuscript, and look forward to the decision.**

**Yours sincerely,**

**Guangxu Ma**

**On behalf of all authors.**
